# The Importance of Polarity in the Evolution of the K^+^ Binding Site of Pyruvate Kinase

**DOI:** 10.3390/ijms151222214

**Published:** 2014-12-02

**Authors:** Leticia Ramírez-Silva, Carlos Guerrero-Mendiola, Nallely Cabrera

**Affiliations:** 1 Departamento de Bioquímica, Facultad de Medicina, Universidad Nacional Autónoma de México, 04510 Distrito Federal, Mexico; E-Mail: carlos@bq.unam.mx; 2 Departamento de Bioquímica, Instituto de Fisiología Celular, Universidad Nacional Autónoma de México, 04510 Distrito Federal, Mexico; E-Mail: ncabrera@ifc.unam.mx

**Keywords:** pyruvate kinase, K^+^, ion selectivity, monovalent cation, hydrophobicity, site-directed mutagenesis

## Abstract

In a previous phylogenetic study of the family of pyruvate kinase, we found one cluster with Glu117 and another with Lys117. Those sequences with Glu117 have Thr113 and are K^+^-dependent, whereas those with Lys117 have Leu113 and are K^+^-independent. The carbonyl oxygen of Thr113 is one of the residues that coordinate K^+^ in the active site. Even though the side chain of Thr113 does not participate in binding K^+^, the strict co-evolution between position 117 and 113 suggests that T113 may be the result of the evolutionary pressure to maintain the selectivity of pyruvate kinase activity for K^+^. Thus, we explored if the replacement of Thr113 by Leu alters the characteristics of the K^+^ binding site. We found that the polarity of the residue 113 is central in the partition of K^+^ into its site and that the substitution of Thr for Leu changes the ion selectivity for the monovalent cation with minor changes in the binding of the substrates. Therefore, Thr113 is instrumental in the selectivity of pyruvate kinase for K^+^.

## 1. Introduction

Rabbit muscle pyruvate kinase (WT-RMPK) was the first enzyme for which an absolute requirement for K^+^ was documented [1]. WT-RMPK can also catalyze in the presence of Rb^+^, NH_4_^+^ and Tl^+^, but the activity is 80%–60% of that observed with K^+^; with Cs^+^, Na^+^ and Li^+^, it is only 9%, 8% and 2%, respectively [2].

**Figure 1:**
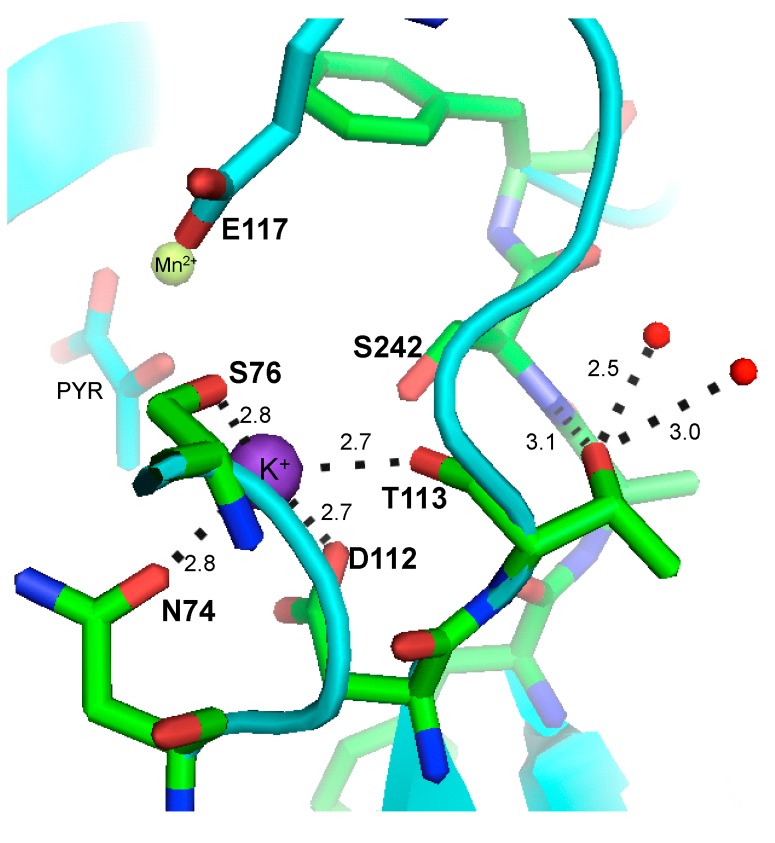
Stick model of the K^+^ binding site of pyruvate kinase from rabbit muscle. The dashed lines from K^+^ (purple sphere) show coordination and distance to the main-chain carbonyl oxygen of T113, O^γ^ of S76, O^δ1^ of N74 and O^δ1^ of D112. The hydrogen bonds formed between the side chain hydroxyl of T113 and NH of Cα of S242 and two water molecules (red spheres) are illustrated. Pyruvate (cyan), Mn^2+^ (lime green) and E117 are also illustrated. This figure was constructed from the coordinates of protein data bank (PDB) 2G50 [10] using the program, PYMOL v0.99.

Up to now, the role of K^+^ in catalysis has not totally been understood, even when it has been extensively studied [3,4,5,6,7]. Recently, it has been shown that K^+^ changes the kinetic mechanism of WT-RMPK, inducing the closure of the active site and promoting a rearrangement of the residues involved in the binding of ADP-Mg [8]. The crystal structures of the enzyme show that K^+^ lies in a site formed by the carbonyl oxygen of T113, O^γ^ of S76, O^δ1^ of N74 and O^δ1^ of D112 [9,10] (Figure 1), a water molecule and a phosphate oxygen of either phosphoenolpyruvate (PEP) [11] or γ-phosphate of ATP [12].

For a long time, it was considered that the dependence of K^+^ was a common feature of all pyruvate kinases (PKs). However, as more enzymes were characterized, it became apparent that the activity of a substantial number of PKs was K^+^-independent. To explore the molecular basis of this behavior, Laughlin and Reed [13] compared the amino acid sequence of WT-RMPK with those of two K^+^-independent bacterial enzymes. The authors found that E117 of WT-RMPK, which is close to the K^+^ binding site, is replaced by Lys in the bacterial enzymes; they constructed the E117K mutant of the rabbit enzyme and found that the mutant was >200-fold more active than the wild-type in the absence of K^+^ and that the activity was not stimulated by monovalent cations.

To ascertain the abundance of K^+^-independent activities, an extensive phylogenetic study of the PK family was performed [14]. Two clusters of about the same number of sequences were clearly separated; one with E117 and the other with K117. Those with E117 exhibit K^+^-dependent activity, whereas those with K117 are K^+^-independent PKs. Ninety seven percent of the PKs that have E117 also have T113, and 97% of the enzymes that have K117 have L113. Laughlin and Reed [13] compared the binding site of K^+^ in WT-RMPK with that of the two bacterial PKs that do not require K^+^ for activity; they found a strict conservation of the residues contributing the K^+^ binding pocket in the three enzymes, except for the substitution of Leu by Thr at residue 113 in the PKs of the bacterial enzymes that do not require monovalent cations for activity. The authors considered that this was a conservative substitution, because the main-chain carbonyl oxygen of T113, and not the O in its side chain, is the K^+^ coordinating moiety (Figure 1). 

The strict co-evolution of the amino acids in positions 117 and 113 in the two clusters of the PK phylogenetic tree [14] suggests that the conservation of T113 in the cluster of K^+^-dependent enzymes is the result of the evolutionary pressure to maintain activities that are strictly dependent on K^+^. Thus, in this work, we explored if, and how, the replacement of Thr of WT-RMPK by Leu in position 113 affects the kinetics of the enzyme for K^+^ and other metal ions. The data show that the substitution affects the kinetics of the monovalent cations and of the substrates, although in a different manner; while in the mutant T113L, the affinity of the monovalent cations is smaller with the higher dehydration energy, the affinity of phosphoenolpyruvate (PEP) and ADP-Mg diminished independently of the size or dehydration energy.

## 2. Results and Discussion

### 2.1. Ion Selectivity of WT-RMPK and T113L-RMPK in 100% Water

**Figure 2:**
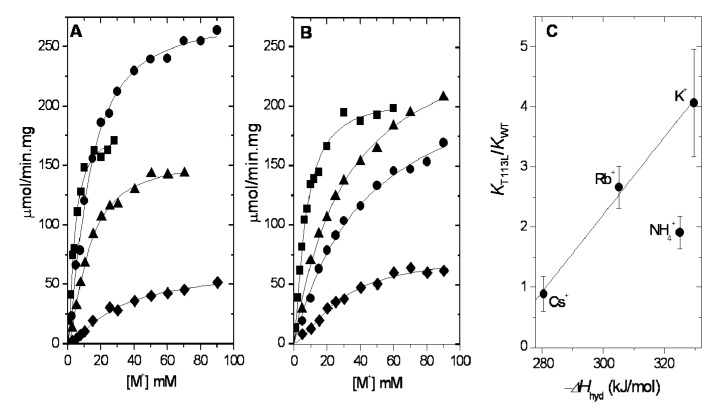
Activation of WT-RMPK (**A**) and T113L-RMPK (**B**) by K^+^ (●), NH_4_^+^ (■), Rb^+^ (▲) or Cs^+^ (♦) at saturating PEP^3−^ and ADP-Mg complex in 100% water; (**C**) *K*_T113L_/*K*_WT_ ratios for the indicated monovalent cations against their heats of dehydration. The activities in the presence of the indicated concentrations of monovalent cations were measured. The mean of two experiments is shown in (**A**) and (**B**). The ratios from (**C**) were calculated from the data of Table S3. *K* represents *K_m_* or *K_0.5_*. Heats of hydration were taken from Marcus [16]. The linear regression (*r* = 0.99) was built taking into account the ratios of alkali metal ions (K^+^, Rb^+^ and Cs^+^) that share basic physicochemical properties [16,17].

We measured and compared the activation of WT-RMPK and T113L-RMPK by different concentrations of K^+^, NH_4_^+^, Rb^+^ and Cs^+^ (Figure 2) in media that contained ten-fold concentrations of the calculate *K_m_* for PEP^3^^−^ and ADP-Mg complex (for the latter data, see Tables S1 and S2). In confirmation of previous results [2], we observed that WT-RMPK reached maximal activation in the presence of K^+^ followed by NH_4_^+^, Rb^+^ and Cs^+^ (Figure 2A); in T113L-RMPK, maximal activation followed the order Rb^+^ > NH_4_^+^ > K^+^ > Cs^+^ (Figure 2B). The experiments were conducted at monovalent concentrations lower than those that induce inhibition (*K_i_* for K^+^ is 142 ± 69 mM) [15]. The kinetic constants of the activation of WT-RMPK and T113L-RMPK by the metal ions are shown in Table S3. It is noted that the *K_m_* or *K_0.5_* for all cations studied, except for Cs^+^, were higher in the mutant than in the WT-RMPK; however, the relative increase in *K_m_* was different for each cation, indicating that T113 may participate in the selectivity of PK for a monovalent cation.

Accordingly, we asked how the change of Thr by Leu could affect the selectivity for the monovalent cations. In search of an explanation, we examined the crystal structure of PK. The data show that the side chain of T113 faces the outside of the active site [9,10] and that its hydroxyl is at hydrogen bond distance of the NH of Cα of S242 and of two water molecules (Figure 1). These water molecules are forming a water network and water-protein interactions with S242 involved in the binding of PEP and with F243, whose phenyl ring orientation flips up in the closure of the lid (see Figure S1). However, it is uncertain if the water network suffers any change by missing the contact with residue 113. Thus, we hypothesized that the substitution of a polar Thr by the hydrophobic Leu, where at least three polar contacts will be missing, could bring about an increase in the hydrophobicity of the active site, thus hindering the partition of the cation into the active site pocket. In this connection, it is relevant to note that differences in the energy of desolvation of K^+^, NH_4_^+^, Rb^+^ and Cs^+^ have been documented [16,18]; and that K^+^ has the highest energy barrier of dehydration. Thus, to examine if there is a correlation between the energy of dehydration of the monovalent cations and their partition into the WT-RMPK and T113L-RMPK, the ratio of the *K_m_* or *K_0.5_* of the mutant and the wild-type enzymes with K^+^, NH_4_^+^, Rb^+^ and Cs^+^ were plotted against the heats of dehydration of the respective cations (Figure 2C). It was found that the affinity of K^+^ in T113L-RMPK was 4.1-times lower than for WT-RMPK. It is important to note that Li^+^ and Na^+^ were not included in this study: this is because in T113L-RMPK, Li^+^ exerts minimal mixed activating and inhibiting effects (0.6 µmol/min·mg), whereas with Na^+^, up to 100 mM, there is only a low non-saturating activation (2.5 µmol/min·mg) (data not shown). On the other hand, for comparative purposes, the effect of NH_4_^+^ was included in the data, particularly because it is a good activator of PK, albeit that it does not have the physicochemical properties of the alkali metal ions.

### 2.2. Ion Selectivity of WT-RMPK and T113L-RMPK with 20% DMSO

**Figure 3:**
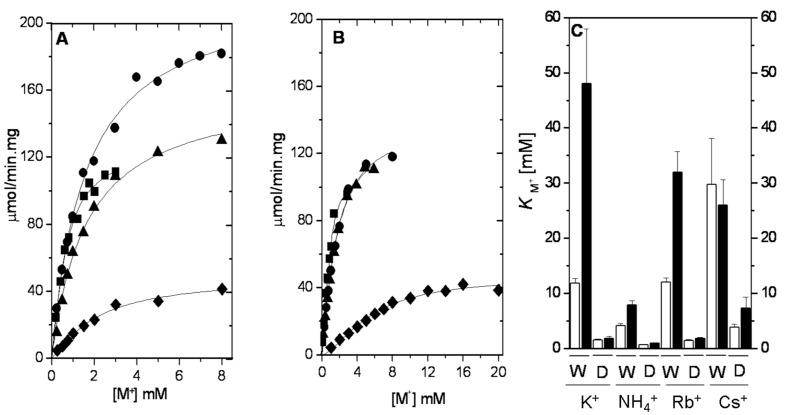
Activation of WT-RMPK (**A**) and T113L-RMPK (**B**) by K^+^ (●), NH_4_^+^ (■), Rb^+^ (▲) or Cs^+^ (♦) at saturating PEP^3−^ and ADP-Mg complex in 20% DMSO; (**C**) *K* of monovalent cations indicated for WT-RMPK (white bars) compared to those of T113L-RMPK (black bars) in 100% water (W) and 20% DMSO (D). The activities in the presence of the indicated concentrations of monovalent cations were measured. The mean of two experiments is shown in (**A**) and (**B**). In plot (**C**), the constants and standard deviations were taken from Tables S3 and S6, where *K* represents *K_m_* or *K_0.5_.*

The data in Figure 2C indicate that the activating effect of metal ions on PK activity could be related to their partition into the catalytic site. To ascertain if this event is indeed related to the ionic selectivity of PK, we determined the effect of K^+^, NH_4_^+^, Rb^+^ and Cs^+^ on the kinetics of WT-RMPK and T113L-RMPK in media with 20% DMSO. This is because it has been well documented that the transfer of relatively polar ligands into relatively hydrophobic protein binding sites is favored by including DMSO in the media [19]. Moreover, it has been shown that the affinity of several enzymes for substrates [20] and cofactors [15] is increased in media with DMSO and that this phenomenon correlates with the partition of the ligands into their binding sites [20]. Co-solvents may also favor the formation and stability of the ligated conformational state of the protein [15,21,22,23,24]. Figure 3 shows the effect of different concentrations of K^+^, NH_4_^+^, Rb^+^ or Cs^+^ on the activity of WT-RMPK (Figure 3A) and that of T113L-RMPK (Figure 3B) in media with 20% DMSO. This was determined at concentrations of PEP^3−^ and ADP-Mg that were 10-times above their *K_m_*_(*app*)_ or *K_0.5_*_(*app*)_ for PEP^3−^ (Tables S4 and S5). In consonance with the reported data [15], PK exhibited a lower activity in 20% DMSO than in water media. However, in comparison to activity in water media, in mixtures with 20% DMSO, maximal activity was obtained with 10-fold lower concentrations of the monovalent cations. The kinetics of the action of the cations in DMSO were studied at concentrations of PEP^3−^ and ADP-Mg that were 10-times above their *K_m_*_(*app*)_ or *K_0.5_*_(*app*)_ for PEP^3−^ (Tables S4 and S5).

The selectivity of the action of monovalent cations in WT-RMPK in media with and without DMSO is very similar (compare data in Figure 2A and Figure 3A). This is in contrast to the effect of the monovalent cations in T113L-RMPK, where it was found that the mutant enzyme, with the exception of Cs^+^, did not show a preference for a particular cation; *i.e.*, the concentration curves for K^+^, NH_4_^+^ and Rb^+^ overlapped (Figure 3B).

The kinetic constants for the action of the monovalent cations in 20% DMSO media for the two enzymes are in Table S6. In both the wild-type and the mutant enzymes, the cation with the highest affinity was NH_4_^+^, which is in agreement with previous reports [25,26,27]. Therefore, it is noteworthy that in conventional water mixtures, WT-RMPK exhibited higher affinities for K^+^ and Rb^+^ than for Cs^+^, whereas the T113L-RMPK mutant showed higher affinities for Cs^+^ and Rb^+^ than for K^+^ (Figure 2B and Table S3). These orders of effectiveness are significantly different in 20% DMSO for T113L-RMPK, where the enzyme exhibited higher affinities for K^+^ and Rb^+^ than for Cs^+^ (Figure 3B). Therefore, the overall data in the presence and absence of 20% DMSO show that the selectivity for monovalent cations is significantly affected by the nature of the residue at position 113. Moreover, the discrimination for K^+^ of T113L-RMPK observed in the water media was abolished when the solvation energy barriers of K^+^ were diminished.

### 2.3. Dehydration Energy of K^+^, NH_4_^+^, Rb^+^ and Cs^+^ and Their Partition into the Catalytic Site of WT-RMPK and T113L-RMPK

**Figure 4:**
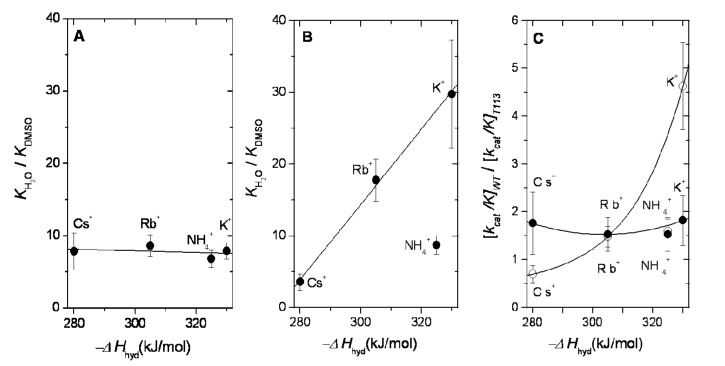
Ratios of *K* of the indicated monovalent cations in water and in 20% DMSO media *versus* their heats of dehydration for WT-RMPK (**A**) and for T113L-RMPK (**B**); In (**C**), the ratios of specificity constants (*k_cat_*/*K*) of the indicated cations in WT-RMPK to those in T113L-RMPK in 100% water (○) and in 20% DMSO (●) are plotted against their heats of dehydration. The ratios of *K* and of specificity constants (*k_cat_*/*K*) were calculated from the data shown in Tables S3 and S6. *K* represents *K_m_* or *K_0.5_.* The correlation coefficients of (**A**) and (**B**) were 0.36 and 0.99, respectively. Data in (○) and in (●) were fitted to the equations *y* = *y*_0_ + Ae^(*x*/t)^ and *y* = A + B*x* + C*x*^2^, respectively.

We explored if the favorable partition of the monovalent cations into their binding site in WT-RMPK and T113L-RMPK in media with 20% DMSO is related to their heat of dehydration. Figure 4A,B depicts the plot of the *K*_(water)_/*K_app_*_(DMSO)_ ratios of K^+^, NH_4_^+^, Rb^+^ or Cs^+^ against their −*∆H*_hyd_ in WT-RMPK and T113L-RMPK, respectively. These ratios indicate that the affinities for K^+^, NH_4_^+^, Rb^+^ and Cs^+^ increase ~8-fold when the enzyme is transferred from 100% water to a medium with 20% DMSO. These *K*_(water)_/*K_app_*_(DMSO)_ ratios do not correlate with their dehydration energy (Figure 4A). In contrast, the affinities of K^+^, Rb^+^ and Cs^+^ for binding into the active site of T113L-RMPK is favored 30-, 18- and 4-fold when the enzyme is transferred from 100% water to a medium with 20% DMSO and nine-fold for NH_4_^+^. This indicates that the *K*_(water)_/*K_app_*_(DMSO)_ ratios were clearly dependent on the dehydration energy; the smaller the cation, the higher the cost to dehydrate it and transfer it into its hydrophobic binding site (Figure 4B).

In sum, even when DMSO favors the partition of the monovalent cations into the active sites of either WT-RMPK or T113L-RMPK, their partition into the wild-type enzyme was about the same, independent of the size of the cation; whereas in the mutant enzyme, it was directly dependent on its size.

### 2.4. Dehydration Energy of K^+^, NH_4_^+^, Rb^+^ and Cs^+^ and Their Specificity for WT-RMPK and T113L-RMPK

The specificity constant, *k_cat_*/*K_m_*, is a useful kinetic value in the identification of the best substrate for a particular enzyme. The values for the two enzymes in 100% water and in 20% DMSO are shown in Tables S3 and S6, respectively. The ratios of the specificity constants (*k_cat_*/*K_m_*) in WT-RMPK to those of T113L-RMPK for K^+^, NH_4_^+^, Rb^+^ and Cs^+^ were 4.6, 1.6, 1.5 and 0.7 in 100% water and 1.8, 1.5, 1.5 and 1.8 in 20% DMSO, respectively. These values represent the extent of preference of WT-RMPK for a monovalent cation compared with T113L-RMPK, the larger the ratio, the more the discrimination of T113L-RMPK for a given monovalent cation. The ratios of specificity constants for each monovalent cation for WT-RMPK and T113L-RMPK in 100% water and in 20% DMSO *versus* their dehydration energy are in Figure 4C. In 100% water, the data indicate that WT-RMPK is about five-fold more specific for K^+^ (with the highest dehydration energy) than T113L-RMPK, whereas the T113L mutant is 1.4-fold more specific for Cs^+^ (with the lowest dehydration energy) than WT-RMPK. In addition, Rb^+^ and NH_4_^+^ (with the intermediate dehydration energies) are about 1.5-fold more specific for WT-RMPK than for T113L-RMPK, either in 100% water or 20% DMSO. In contrast, when DMSO exerts its effect in partitioning monovalent cations into their binding site, WT-RMPK is about 1.8-fold more specific for K^+^ and Cs^+^, respectively, than for T113L-RMPK. These data indicate that T113L-RMPK discriminates K^+^ in 100% water; and that when the partition of the cation into its binding site is favored by DMSO, the discrimination is almost abolished. Therefore, the substitution of Thr by Leu is instrumental in the discrimination of T113L-RMPK for K^+^.

### 2.5. PEP and ADP-Mg Binding Sites

**Figure 5:**
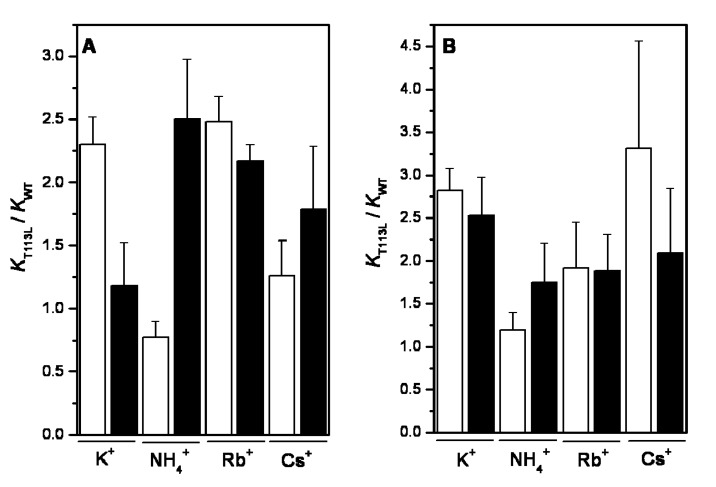
Ratios of *K* of PEP^3−^ for T113L-RMPK and WT-RMPK (**A**) and ratios of ADP-Mg for T113L-RMPK and WT-RMPK (**B**) in 100% of water (white bars) and in 20% DMSO (black bars) activated by the indicated monovalent cations. *K* of PEP^3−^ for both enzymes in 100% water and in 20% DMSO were taken from Tables S1 and S4, respectively; *K* of the ADP-Mg complex for both enzymes in 100% water and in 20% DMSO was taken from Tables S2 and S5, respectively. *K* represents *K_m_* or *K_0.5_.* For the experiments carried out in 100% water, NH_4_^+^ was 30 and 60 mM for WT-RMPK and for T113L-RMPK, respectively; the concentration of K^+^, Rb^+^ and Cs^+^ was 90 mM for both enzymes. For the experiments carried out in 20% DMSO, K^+^, NH_4_^+^ and Rb^+^ were 15, 9 and 17 mM, respectively, for both enzymes; the concentration of Cs^+^ was 85 and 40 mM for WT-RMPK and T113L-RMPK, respectively.

In order to explore if the substitution of Thr113 by Leu113 affects the binding of the substrates, we compared the ratios *K*_T113L_/*K*_WT_ of PEP^3−^ (Figure 5A) in the presence of saturating concentrations of ADP-Mg, K^+^, NH_4_^+^, Rb^+^ and Cs^+^. A similar plot was built for ADP-Mg in the presence of saturating concentrations of PEP^3−^, K^+^, NH_4_^+^, Rb^+^ and Cs^+^ (Figure 5B). The experiments were carried out in conventional aqueous media and in mixtures with DMSO. The results showed that the inclusion of the hydrophobic Leu in the active site with K^+^, NH_4_^+^, Rb^+^ and Cs^+^ decreased the affinity of the substrates (*K*_T113L_/*K*_WT_ > 1) to a similar extent in both water medium or medium with 20% DMSO; *i.e.*, the ratios *K*_T113L_/*K*_WT_ of the affinity for PEP^3−^ varied from 0.8- to 2.5-fold and those for ADP-Mg varied from 1.2- to 3.3-fold. This effect on the affinities for both substrates did not correlate with the heats of dehydration or the size of the respective monovalent cations. Therefore, the binding of the substrates into T113L-RMPK is independent of the restriction imposed to the monovalent cation binding site.

In addition to the co-evolution of T113 with E117, the phylogenetic analysis of K^+^-dependent PKs shows that residues K114 and T120 covariate 89% and 81%, respectively, with E117 [14]. In the K^+^-independent PKs, Q114 and (I, L, V) 120 covariate 82% and 93%, respectively with K117. Therefore mutants K114Q and T120L of RMPK were also constructed and studied. Mutant K114Q preserved ion selectivity and kinetic constants for monovalent cations similar to those exhibited by WT-RMPK. Only minor changes were found in the kinetic parameters for the substrates. On the other hand, mutant T120L was poorly expressed, unstable and did not exhibit any activity in the free salt form. Albeit having K^+^ or NH_4_^+^, *V_max_* of the T120L mutant was 400-fold lower than that of WT-RMPK, with similar affinities for the substrates (data not shown). Residue 120 is part of the hinge that closes and opens the lid (domain B) over the active site (domain A). In the open conformation, O^γ^ of Thr120 forms hydrogen bonds with main-chain NH of Lys206 and with a water molecule. In the closed conformation, none of these interactions are present. Lys206 moves 10.4 Å when the lid is closed to coordinate the ribose of the nucleotide in the active site. Therefore, it is likely that the movement of the lid is altered in the mutant, T120L.

## 3. Materials and Methods

### 3.1. Mutagenesis of T113L Pyruvate Kinase (T113L-RMPK)

The T113L-RMPK was constructed using the WT-RMPK plasmid by means of polymerase chain reaction (PCR) using the Expand High Fidelity PCR System (Boehringer, Ingelheim am Rhein, Germany). The mutagenic oligonucleotides were 5'-GCTCTGGACCTGAAGGGACC-3' and 5'-GGTCCCTTCAGGTCCAGAGC-3', and the external oligonucleotides were 5'-TAATCATCCGGCTCGTATAATGTG-3' and 5'-GGCTGAAAATCTTCTCTCATCCGC-3'. The mutation was introduced as follows: 5 min at 94 °C, 30 cycles for 3 min at 94 °C, 1 min at 67 °C and 1 min at 72 °C; and the extension incubation for 10 min at 72 °C. The PCR product was cloned in the pTrc99A vector after digestion with EcoRI and sequenced completely. The gene with the appropriate mutation was introduced by transformation into an *E. coli* strain devoid of PK (PB25) [28].

### 3.2. Cell Growth and Purification of the T113L Mutant

LB medium containing 100 µg/mL ampicillin, 50 µg/mL kanamycin and 30 µg/mL chloramphenicol was inoculated with PB25-T113L. Expression was induced with 1 mM isopropyl 1-thio-β-d-galactopyranoside at an A_600_ of 0.6. The enzyme was purified as in [8]. The T113L-WTPK was 95% pure, as indicated by SDS-PAGE and Coomassie staining.

### 3.3. Assays of Pyruvate Kinase Activity

WT-RMPK and LDH were obtained as ammonium sulfate suspensions from Roche Applied Science. Ammonium sulfate-free enzymes were obtained as in [29]. The cyclohexylammonium salts of ADP and PEP were used. NADH sodium salt was converted to the cyclohexylammonium salt following the protocol of Sigma-Aldrich (St. Louis, MO, USA). Tetramethylammonium hydroxide was prepared as in [8]. Contaminating NH_4_^+^, Na^+^ and K^+^ in reaction mixtures were below the detection limit (10 µM), as shown elsewhere [29]. The formation of pyruvate was measured spectrophotometrically as in [30].

The reaction mixtures, either in conventional aqueous media (100% water) or composed by 20% (*w*/*v*) DMSO, contained 25 mM HEPES-tetramethylammonium hydroxide, pH 7.4, and the concentrations of cations (K^+^, NH_4_^+^, Rb^+^ and Cs^+^) indicated under the “Results and Discussion”. The ADP-Mg complexes and free Mg^2+^ concentrations were calculated using the software, CHELATOR [31]. Ionized PEP concentrations were calculated considering a p*K* value of 6.3 [32]. Since dielectric constants in conventional aqueous medium and in mixtures with 20% DMSO (*w*/*v*) are very similar (78.36 and 77.45, respectively) [33], the concentrations for ADP-Mg complexes and PEP^3−^ in media with 20% DMSO were calculated as described above. For the experiments carried out in 100% water with saturating concentrations of the substrates, the fixed concentrations of PEP^3−^ for WT-RMPK were 0.46 mM for all monovalent cations and for T113L-RMPK were 1.19, 0.46, 0.64 and 0.34 mM, with K^+^, NH_4_^+^, Rb^+^ and Cs^+^, respectively. The fixed concentrations for the ADP-Mg complex for WT-RMPK were 3.7 mM for K^+^ and Rb^+^ and 2.42 and 2.93 mM for NH_4_^+^ and Cs^+^, respectively. For T113L-RMPK, ADP-Mg concentrations were 12.1, 2.96, 7.26 and 10.28 mM for K^+^, NH_4_^+^, Rb^+^ and Cs^+^, respectively. For the experiments carried out in 20% DMSO with saturating concentrations of the substrates, the fixed concentrations of PEP^3−^ were 0.46 mM for all monovalent cations and for T113L-RMPK were 0.23, 0.11, 0.129 and 0.23 mM for K^+^, NH_4_^+^, Rb^+^ and Cs^+^, respectively. The fixed concentrations for the ADP-Mg complex for WT-RMPK were 1.06, 0.73, 0.86 and 0.46 mM for K^+^, Rb^+^, NH_4_^+^ and Cs^+^, respectively, and for T113L-RMPK were 2.93, 1.32, 1.65 and 0.99 mM for K^+^, NH_4_^+^, Rb^+^ and Cs^+^, respectively. HEPES was added to achieve a constant ionic strength of 0.223 M using the software, Maxchelator (http://maxchelator.stanford.edu), when different ligand concentrations (PEP, ADP, MgCl_2_ and monovalent cations) were used. It was checked that the concentration of LDH added to the reaction mixture (10 and 30 µg/mL for water media and 20% DMSO mixtures, respectively) sufficed to trap all of the formed pyruvate, *i.e.*, the specific activity was not increased by a 5-fold increase in the concentration of LDH. It is noted that divalent metal ions are essential for phospho transfer during the catalytic cycle of PKs and that Mg^2+^ is considered to be the physiologically relevant divalent metal ion. Therefore, all of the experiments were carried out in the presence of a non-inhibitory free Mg^2+^ concentration (2.96 mM). Activity was initiated with 0.1 µg/mL of WT-RMPK or T113L-RMPK. The temperature was 25 °C. Kinetic data were fitted either to the Michaelis–Menten equation (*v = Vmax ∙ S*/*K_m_* + *S*) or to the Hill equation (*v = Vmax* ∙ *S^n^*/*K_0.5_^n^* + *S^n^*) using Origin version 7.0. The standard deviations from two experiments are shown in the tables of Supplemental Data. We computed the standard deviations of the ratios shown in Figure 2C, Figure 4 and Figure 5 as provided by [34].

## 4. Conclusions

The goal of this work was to understand why T113 is a conserved residue in the family of the K^+^-dependent PKs [14]. During evolution, Thr was conserved to be part of the monovalent cation binding site, even though K^+^ is coordinated by its carbonyl oxygen and not by the oxygen of its side chain, which, in fact, is oriented toward the outside of the monovalent binding site. We found that the polarity of the residue 113 is determinant in the partition of K^+^ into its site. We also found that the substitution of Thr by Leu into the active site of PK locally altered the binding site for the monovalent cation with minor changes in the binding site for PEP^3−^ and ADP-Mg. Therefore, T113 was conserved, because it participates in the selectivity for K^+^ in the K^+^-dependent family of PK.

## Supplementary Materials

Supplementary materials can be found at http://www.mdpi.com/1422-0067/15/12/22214/s1.
